# Neurometabolic signatures of gastrointestinal symptoms in the insula of Crohn’s disease patients: explorative findings from a 7T MRS study

**DOI:** 10.3389/fnhum.2025.1620488

**Published:** 2025-11-20

**Authors:** Marja-Lisa Berthold, Hanna Hartmann, Ezequiel Farrher, Markus Zimmermann, Julius Jaeger, N. Jon Shah, Kai Markus Schneider, Irene Neuner, Ravichandran Rajkumar

**Affiliations:** 1 Department of Psychiatry, Psychotherapy and Psychosomatics, University Hospital Aachen, RWTH Aachen University, Aachen, Germany; 2 Institute of Neuroscience and Medicine 4 (NM-4), Forschungszentrum Jülich GmbH, Jülich, Germany; 3 Department of Diagnostic and Interventional Radiology, University Hospital Düsseldorf, Heinrich Heine Universität Düsseldorf, Düsseldorf, Germany; 4 Department of Gastroenterology, Metabolic Diseases and Internal Intensive Care Medicine, University Hospital Aachen, RWTH Aachen University, Aachen, Germany; 5 Department of Medicine 1, University Hospital Carl Gustav Carus Dresden, Technische Universität Dresden, Dresden, Germany; 6 Else Kröner Fresenius Center for Digital Health, Technische Universität Dresden, Dresden, Germany; 7 Center for Regenerative Therapies Dresden (CRTD), Technische Universität Dresden, Dresden, Germany; 8 JARA-BRAIN, Aachen, Germany; 9 Department of Neurology, University Hospital Aachen, RWTH Aachen University, Aachen, Germany; 10 Institute of Neuroscience and Medicine 11 (INM-11), Forschungszentrum Jülich GmbH, Jülich, Germany

**Keywords:** Crohn’s disease, insular cortex, pain, magnetic resonance imaging, proton magnetic resonance spectroscopy

## Abstract

**Background:**

The bidirectional communication between the brain and gut in Crohn’s disease is increasingly acknowledged, highlighting how gut inflammation can influence brain function and psychological health, and vice versa, through the gut-brain axis. The insula is critical for processing pain, its emotional evaluation, and for regulating neurometabolites involved in these processes. The role of insular neurometabolites in gastrointestinal symptoms, particularly pain, in Crohn’s disease patients, however, is not well-understood, highlighting the need for further investigation. Therefore, this study aims to enhance our understanding of the connection between Crohn’s disease and brain function by investigating neurometabolic profiles in the insula of patients with Crohn’s disease.

**Methods:**

In this study, 7 Tesla proton magnetic resonance spectroscopy (^1^H-MRS) was utilized to examine the left insular cortex in 14 individuals with Crohn’s disease and 14 age- and gender-matched healthy controls during resting state. Participants also completed neuropsychological evaluations, including the Gastrointestinal Symptom Rating Scale (GSRS) and the Pain Catastrophizing Scale (PCS).

**Results:**

No significant differences were found in the absolute concentrations of insular neurometabolites between Crohn’s disease patients and healthy controls. However, in patients with Crohn’s disease, GSRS scores were negatively correlated with the neurometabolites aspartate (Asp) and N-acetylaspartylglutamate (NAAG) in the insula. Furthermore, significant positive correlations were observed between scores on the PCS magnification subscale and concentrations of neurometabolites—namely glutamine (Gln) and the combined glutamate and glutamine signal (Glx) —as measured by (^1^H-MRS).

**Conclusion:**

The neurometabolic alterations observed in the insular cortex of Crohn’s disease patients suggest increased insular activity, which may enhance interoceptive awareness and pain sensitivity, potentially contributing to heightened pain catastrophizing.

## Graphical Abstract

**Figure:**
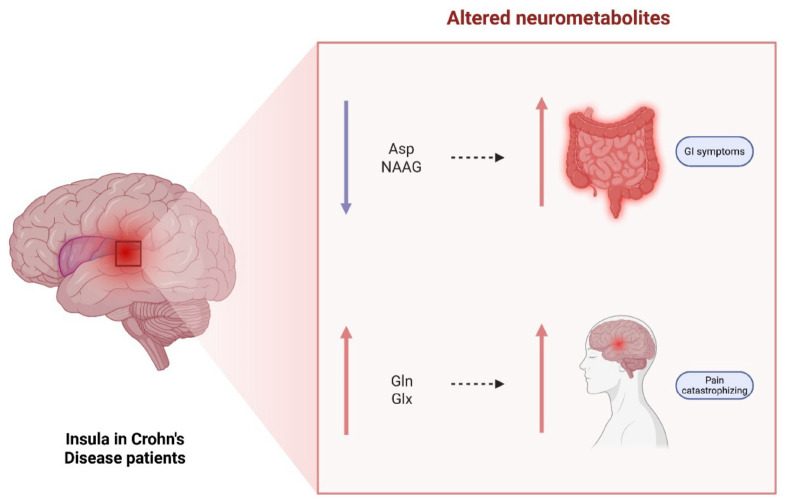
Visual abstract for neurometabolic signatures of gastrointestinal symptoms in the insula of Crohn’s disease patients: explorative findings from a 7T MRS study. Created in BioRender. Berthold, M. (2025) https://BioRender.com/8jnxlln. Asp, aspartate; NAAG, *N*-acetylaspartylglutamate; Gln, glutamine; Glx, glutamate + glutamine.

## Introduction

Crohn’s disease is a type of inflammatory bowel disease (IBD) characterized by inflammation and irritation in the digestive tract. Crohn’s disease profoundly impacts various aspects of patients’ lives, with pain being a notable symptom (Bielefeldt et al., 2009). The pathogenesis of Crohn’s disease involves a complex interplay between genetic predispositions and environmental factors, including medications, diet, and psychological stress, alongside microbial dysbiosis within the gut (Ananthakrishnan et al., 2018; Bitton et al., 2008; Lopetuso et al., 2017; Schneider et al., 2023; Shaw et al., 2010; Stolzer et al., 2020; Zhang and Li, 2014). Dysbiosis causes localized intestinal inflammation, which can become systemic, influencing brain function via pathways (Bonaz and Bernstein, 2013) such as the nociceptive system and the autonomic nervous system (ANS), notably through the vagus nerve (Masanetz et al., 2022; Wan et al., 1994). This process then potentially leads to increased sensitivity to nociceptive stimuli mediated by insula activation (Harrison et al., 2009, 2015, 2016). Both inflammatory signals and pain are known to stimulate the insula, which then enhances sensitivity to pain (Gogolla, 2021; Karshikoff et al., 2016). The insula, pivotal in interoception, acts with the amygdala to convert noxious stimuli into the sensation of pain (Gelebart et al., 2023). Painful stimuli are initially processed by the posterior granular insula, which assesses the sensory qualities of the nociceptive signal, including its quality and intensity. They are then relayed through the middle dysgranular insula to the anterior insula, where they integrate with cognitive and emotional components to form a conscious perception of pain. Simultaneous activation of both sides of the anterior insula then contributes to the subjective experience and evaluation of pain (Benarroch, 2019; Gelebart et al., 2023; Uddin, 2015). Thus, it is suggested that the insula plays a crucial role in the neuropathology of pain in Crohn’s disease and is thereby also involved in the gastrointestinal symptoms.

Prior studies have explored the influence of various neurometabolites on pain perception in the context of both acute pain and chronic pain syndromes using hydrogen proton magnetic resonance spectroscopy (^1^H-MRS), which enables *in vivo* assessment of various neurometabolites in the brain. Several studies have indicated that during episodes of acute pain, the concentrations of glutamate (Glu), glutamine (Gln), and glutamate + glutamine (Glx) rise, while the concentrations of *myo*-inositol (Ins) fall within the insula and the anterior cingulate cortex (ACC) (Gutzeit et al., 2011, 2013; Jung et al., 2021; Mullins et al., 2005). Additionally, an association has been demonstrated between Gln levels and the subjective experience of pain intensity (Mullins et al., 2005). Similarly, studies on chronic pain have identified associations between pain perception and insular levels of Glu, Gln, and phosphocreatine (PCr) (Harfeldt et al., 2018; Harris et al., 2008; Jung et al., 2019, 2021).

In this context, ^1^H-MRS studies have also been used to investigate metabolic changes in different brain regions of patients with Crohn’s disease (Kong et al., 2022; Lv et al., 2018; Wang et al., 2023). However, to the best of our knowledge, research into the neurometabolic basis of pain perception in Crohn’s disease patients, specifically within the insula, remains sparse.

Considering the established link between the insula and pain processing, this study aims to leverage single-voxel ^1^H-MRS at 7 Tesla to investigate potential differences in neurometabolite concentrations within the insula between Crohn’s disease patients and healthy controls. Additionally, it seeks to examine how alterations in these neurometabolites may influence symptomatology, particularly pain processing and psychological factors, in Crohn’s disease.

## Materials and methods

### Participants

Fourteen Crohn’s Disease patients (age 29 ± 7, 10 males) and 14 age and gender-matched healthy controls (age 26 ± 4) between the ages of 18 and 40 were included in this study. The participants with Crohn’s disease were enrolled from the Clinic for Gastroenterology, Metabolic Diseases and Internal Intensive Care Medicine of the University Hospital RWTH Aachen, Germany, where their Crohn’s disease diagnosis was established based on the established clinical guidelines (colonoscopy and histology). Other inclusion criteria for both the Crohn’s disease group and the healthy controls were right-handedness, the absence of underlying psychiatric conditions, and no contraindications for 7T MRI.

Right-handedness was evaluated using the Edinburgh Handedness Inventory (EHI), while the German version 6.0.0 of the Mini International Neuropsychiatric Interview (MINI) was performed to confirm that no psychiatric conditions were present in the participants (Oldfield, 1971; Sheehan et al., 1998). The Ethics Committee of the Medical Faculty of RWTH Aachen University granted approval for this study. All methods were carried out according to the relevant guidelines and regulations. Prior written consent was given by all volunteers, and they were remunerated for their contribution to the research.

### Gastrointestinal Symptom Rating Scale

The 15-item German version of the Gastrointestinal Symptom Rating Scale (GSRS) licensed from Astrazeneca AB (Sweden) was used. It includes five subscales: reflux (Cronbach’s α = 0.72), abdominal pain (α = 0.43), indigestion (α = 0.79), diarrhea (α = 0.84) and constipation (α = 0.81) (Kulich et al., 2008). The answers are assessed on a seven-point Likert scale, ranging from 1 (no symptoms) to 7 (very strong symptoms).

### Pain Catastrophizing Scale

The German version of the Pain Catastrophizing Scale (PCS) with 13 items was used (Cronbach’s α = 0.92) (Wheeler et al., 2019). The scale measures three items in subscales: rumination, helplessness and magnification. The rumination subscale includes four items (α = 0.89), the helplessness subscale includes six items (α = 0.88) and the magnification scale includes three items (α = 0.77) (Wheeler et al., 2019). Participants had to answer the questions on a five-point Likert scale, with 0 being strong disagreement and 4 being total agreement.

### Magnetic resonance spectroscopy

The MR data were acquired by utilizing a 7T Magnetom Terra scanner (Siemens Healthineers, Erlangen, Germany), equipped with a single transmit (Tx) and a 32-channel receive (Rx) head coil from Nova Medical (Wilmington, MA, United States). Structural images were obtained using a T1-weighted MP2RAGE sequence, with the following protocol parameters: inversion image 1 (INV1), inversion time (TI) = 840 ms, flip angle (FA) = 4°, INV2 TI = 2370 ms, FA = 5°, echo time (TE) = 1.99 ms; repetition time (TR) = 4,500 ms, image matrix size = 320 × 300 × 208, voxel size = 0.75^3^ mm.

**FIGURE 1:**
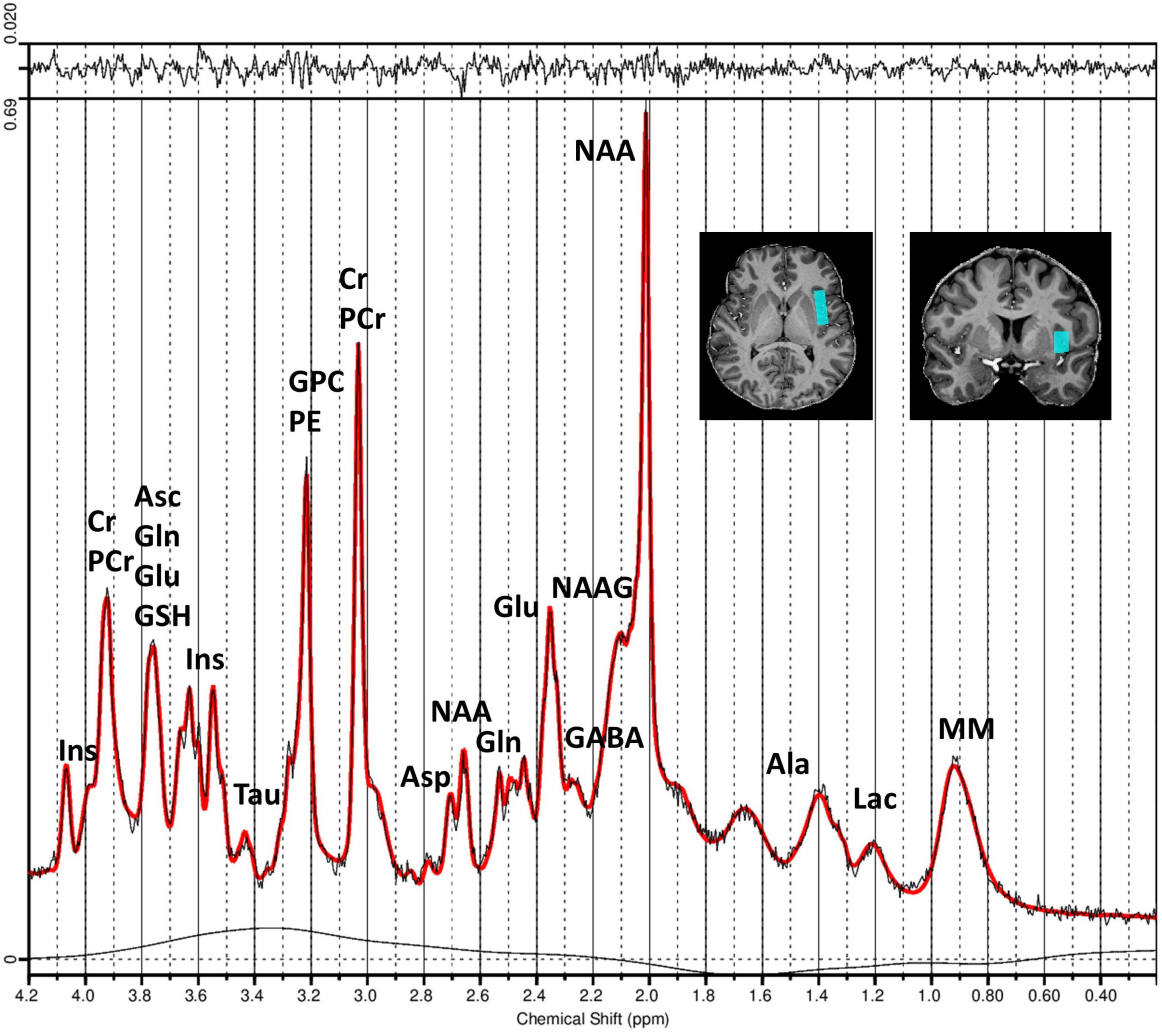
Measured and fitted ^1^H-MRS spectra of the left insula generated by LCModel software. Spectra were obtained using the STEAM sequence (Tkác et al., 1999, 2009) with TE = 4.6 ms, TM = 28 ms, TR = 8,200 ms, and 64 averages. The figure illustrates the obtained MR spectrum (black line) and the fitted spectrum (red line) processed using LCModel software. The residual signal is displayed at the top of the plot, and the chemical shift values are displayed in parts per million (ppm) along the x-axis. The positioning of the MRS voxel within the left insula region is shown in coronal and axial slices of the structural MR image from an exemplary subject (top right). Peaks corresponding to different metabolite concentrations are labeled. Asp, aspartate; Cr, creatine; PCr, phosphocreatine; GABA, gamma-aminobutyric acid; Gln, glutamine; Glu, glutamate; GPC, glycerylphosphorylcholine; GSH, glutathione; Ins, myo-Inositol; Lac, lactate; NAA, *N*-acetylaspartate; NAAG, *N*-acetylaspartylglutamate; Tau, taurine; PE, phosphoethanolamine; Asc, ascorbate; Ala, alanine; MM, macromolecules.

Prior to the acquisition of ^1^H-MRS data, first- and second-order B0 shimming within the ^1^H-MRS voxel of interest was optimized using the fast automatic shimming technique by mapping along projections (FASTESTMAP) (Gruetter and Tkác, 2000). Spectra of the left insula were acquired using the stimulated echo acquisition mode (STEAM) sequence (Tkác et al., 1999, 2009), using an ultra-short TE = 4.6 ms, a mixing time (TM) = 28 ms, TR = 8,200 ms, and 64 averages. The voxel dimensions were set to 34 mm (AP) × 11 mm (RL) × 16 mm (FH). The radiofrequency (RF) pulse was centered at 3.0 ppm, with a receive bandwidth = 6,000 Hz and a vector size = 2,048. The RF power calibration was performed individually for each participant (Deelchand et al., 2015). The acquisition sequence also included water suppression (VAPOR) and outer-volume suppression (OVS) modules (Tkác et al., 1999, 2001). To facilitate eddy-current correction and metabolite concentration quantification, two extra averages were acquired without the VAPOR RF pulses to record the water peak. The single-voxel ^1^H-MRS acquisition was localized to the left insular cortex. Voxel placement was performed manually for each participant using high-resolution T1-weighted anatomical images as guidance. The voxel was positioned in the anterior and posterior left insula and carefully adjusted in the axial, coronal, and sagittal planes to ensure consistent medial-lateral centering within the insular cortex, while avoiding partial volume contamination from adjacent white matter, cerebrospinal fluid, the operculum, and the lentiform nucleus. All voxel placements were reviewed and confirmed by experienced physicians and trained MRS operators to ensure anatomical consistency across participants. An illustration of voxel positioning is provided in Figure 1.

### Spectral data analysis

Hydrogen proton magnetic resonance spectroscopy preprocessing was conducted using the FID-A package (Simpson et al., 2017), available for MATLAB (Version 2022a), and included automatic detection and removal of motion corrupted spectra (Simpson et al., 2017) as well as phase and frequency drifts correction of individual scans via spectral registration in the frequency domain (Near et al., 2015). Subsequent spectral fitting was executed using LCModel (version 6.3-1R) with the water scaling and eddy current correction options enabled. Spectral fitting was conducted across the chemical shift range of 0.2–4.2 ppm. VeSPA, customized for the STEAM sequence, was utilized to create the basis metabolite set used in the LCModel (Soher et al., 2023). The basis set incorporated ideal pulses, actual sequence timings, chemical shifts and J-coupling constants along with nineteen metabolites: aspartate (Asp), Ins, Glu, Gln, GABA, alanine (Ala), ascorbate (Asc), creatine (Cr), glucose (Glc), glutathione (GSH), glycerophosphorylcholine (GPC), lactate (Lac), N-acetylaspartate (NAA), N-acetylaspartylglutamate (NAAG), PCr, phosphorylcholine (PCh), phosphorylethanolamine (PE), *scyllo*-inositol (Scyllo) and taurine (Tau). An additional macromolecular (MM) spectrum, acquired with STEAM and similar protocol parameters at 7T (TE = 6 ms) was also incorporated in the basis set (MM Consensus Data Collection repository^1^). LCModel outputs provided the estimated metabolite concentrations alongside the Cramér-Rao Lower Bounds (CRLB). Metabolites were included in the analysis only if their mean CRLB across participants was below 50%. No individual subject values were excluded solely on the basis of a CRLB > 50%. The metabolite concentrations of total NAA (NAA + NAAG), the Gln + Glu complex (Glx), and the excitation-inhibition ratio (Glx/GABA) were also computed and considered in the subsequent analysis. Metabolite reliability was evaluated based on the distribution of absolute CRLB values, calculated as the product of the metabolite concentration and its relative CRLB reported by LCModel [i.e., absolute CRLB = concentration × (CRLB%/100)] (Kreis, 2016). Outliers were identified as absolute CRLB values exceeding the cohort-specific mean ± 2 standard deviations (calculated separately for controls and Crohn’s disease patients). This cohort-specific approach was chosen to avoid bias from group-wise variability and ensures that the threshold for outlier detection reflects the distribution within each population.

The NAA-based signal-to-noise ratio (SNR) was calculated using the op_getSNR function from the FID-A toolbox. Specifically, SNR is defined as the difference between the NAA peak amplitude (within 1.8–2.2 ppm) and the mean signal at the noise region, divided by the standard deviation of the noise region. The noise was estimated from the real part of the spectrum in the 8–14 ppm range, where no metabolite signals are present. A second-order polynomial fit was applied to remove any residual baseline trends in the noise region before computing the noise standard deviation. This approach provides a robust estimate of spectral quality by accounting for potential DC offsets and baseline curvature.

Metabolite quantification followed the approach outlined by Gasparovic et al. (2006), using the relaxation functions tailored for the STEAM sequence (Knight-Scott and Li, 1997). T1 and T2 relaxation time constants for the water signal were obtained from previous publications (Bartha et al., 2002; Marjańska et al., 2017; Marjańska and Terpstra, 2021; Rooney et al., 2007): T1 = 2,132 ms (GM), 1,220 ms (WM), and 4,425 ms (CSF); T2 = 50 ms (GM), 55 ms (WM), and 141 ms (CSF). Since the subjects were relatively young, age-related changes in the relaxation times were not considered (Marjańska et al., 2013). Given the ultra-short TE and long TR of the used STEAM sequence, the signal relaxation of neurometabolites was neglected. To address the issue of partial volume from various tissue types within the voxel of interest, MR structural images were segmented into GM, WM, and CSF using the tool FAST (Zhang et al., 2001), whereas the tool FIRST (Jenkinson et al., 2012; Patenaude et al., 2011) was used for the segmentation of subcortical structures, both tools available in the toolkit FSL (FMRIB Software Library v6.0.3). Subsequently, the relative volumes of these tissues within the MRS voxel were precisely estimated and used in the quantification.

### Statistical analysis

All statistical analyses were performed using the MATLAB (R2022a) software package. The group differences in metabolite concentrations between healthy controls and Crohn’s disease patients were tested using regression analysis. Since the sample size was low and did not meet the assumptions of parametric analysis, a non-parametric approach was applied. The concentrations of metabolites were rank transformed across both the groups using the *tiedrank* function in MATLAB to mitigate the effects of non-normality. The rank-transformed concentration values were then used to perform regression analyses to test for differences between the two groups while controlling for age and gender as covariates. For each metabolite, a linear regression analysis was performed using the rank-transformed metabolites concentrations as the dependent variable, and “Group” (Healthy vs. Crohn), “Age,” and “Gender” were independent variables (rank-transformed metabolites concentrations ∼ Group + Age + Gender). The *fitlm* function in MATLAB was used to fit linear models. The estimated coefficients for the “Group” variable provided the basis for comparison between healthy controls and Crohn’s disease patients.

### Multicollinearity assessment

To evaluate the presence of multicollinearity, which could influence the regression estimates, the variance inflation factor (VIF) was calculated for age against all other metabolite concentrations. The VIF values showed moderate correlation (range of VIF 3.46–4.09), suggesting that multicollinearity did not affect the analyses.

### Correlation analysis with GSRS and PCS

To explore associations between gastrointestinal symptoms measured via GSRS and PCS in Crohn’s disease patients, a correlation analysis including sub scores and neurometabolite concentrations was conducted. Spearman’s correlation coefficients were computed with a significance level of 5%. In all the correlation analyses, the family-wise error rate (FWER) due to multiple comparisons was controlled for using a permutation test (Groppe et al., 2011). A thousand permutations were performed for each comparison (correlation), and the *p*-value was adjusted using the “max statistics” method (Groppe et al., 2011).

## Results

### Participants

The study included 14 patients with Crohn’s disease and 14 healthy controls matched for age and gender. The majority of patients received pharmacological treatment for the management of Crohn’s disease. Specifically, 71.4% (*N* = 10) were treated with biologics, 21.4% (*N* = 3) with cytostatic or cytotoxic immunosuppressants, and 7.1% (*N* = 1) with glucocorticoids (Table 1). The mean GSRS and PCS scores for Crohn’s disease patients were 1.91 (± 0.77) and 11.08 (± 7.59), respectively (Table 2).

**TABLE 1:** Demographics and medication details of participants.

Characteristics	Crohn’s disease patients (*n* = 14)	Healthy controls (*n* = 14)
Demographics		
Sex (male/female)	10/4	10/4
Age (years)	29.07 ± 7.36	26.07 ± 3.63
Current therapy		
Biologicals	10	–
Cytostatic/-toxic immunosuppressants	3	–
Glucocorticoids	1	–
Cholechalciferol (vit D3)	7	–
Vitamin B12	1	–
Folic acid	2	–
Iron	3	–
Zinc	1	–
Other	5	–

**TABLE 2:** GSRS and PCS scores of participants.

Characteristics	Crohn’s disease patients (*n* = 13)	
	Mean	Standard deviation
**Neuropsychological test scores**		
**GSRS**		
Total	1.91	0.77
Reflux	2	1.43
Abdominal pain	1.83	0.82
Indigestion	2.22	0.85
Diarrhea	1.53	0.76
Constipation	1.64	0.78
**PCS**		
Total	11.08	7.59
Helplessness	3.38	3.25
Magnification	2.69	1.80
Rumination	5	3.51

GSRS, Gastrointestinal Symptom Rating Scale; PCS, Pain Catastrophizing Scale.

### ^1^H-MRS results

Neurometabolite concentrations were assessed in the left anterior and posterior insula of both the Crohn’s disease patients and the healthy controls. The voxel placement in the left insula and the corrected MR spectrum are presented in Figure 1. Most metabolite concentrations in one healthy control and one Crohn’s disease subject exhibited absolute CRLB values exceeding twice the standard deviation of the group mean (Supplementary Figure 1). As such, both subjects were excluded from further analyses. Spectral quality was assessed using the LCModel-derived SNR and the NAA-based SNR computed from the water-suppressed spectra. NAA SNR was evaluated for both the individual transients and the averaged spectrum of each subject to ensure consistency and reliability across participants. Descriptive statistics (mean ± SD) for both SNR metrics are reported separately for each cohort in Table 3. To control for quality, spectra were flagged as low quality if either SNR metric fell more than two standard deviations below the group mean (Supplementary Figure 2). Importantly, no subject met this exclusion criterion, confirming the overall high and consistent spectral quality across both groups. Group-level summary statistics are reported in Table 3 for both healthy controls and Crohn’s disease patients. All individual spectra were visually inspected following motion artifact rejection and spectral alignment. After averaging, the LCModel-fitted spectra, residuals, and baseline functions were reviewed for each participant to ensure adequate spectral quality, proper metabolite fitting, and absence of major distortions or modeling artifacts. The average unsuppressed water linewidth (FWHM) is reported in Table 3. Linewidths were comparable across groups, and all fell within the acceptable range for reliable spectral quantification at 7T (Henning, 2018; Öz et al., 2021). To evaluate the reliability of metabolite quantification, pairwise correlation coefficients between fitted metabolites were extracted from the LCModel output using the LPRINT = 6 flag, which generates the full correlation matrix for each spectrum. These coefficients were used to identify strongly anti-correlated metabolite pairs (e.g., r < −0.7), which may reflect poor separation or fitting ambiguity. Based on this analysis, Cr and PCr, which exhibited high negative correlations in several subjects, were excluded from individual analyses. In contrast, NAA and NAAG, as well as Glu and Gln, showed consistently low correlation coefficients, justifying their reporting as separate metabolites.

**TABLE 3:** Neurometabolic profiles of participants.

Characteristics	Crohn’s disease patients (*n* = 13)			Healthy controls (*n* = 13)		
	Mean	Standard deviation	CRLB (%)	Mean	Standard deviation	CRLB (%)
**Demographics**						
Sex (male/female)	9/4	–	–	9/4	–	–
Age (years)	28.38	7.18	–	26.31	3.66	–
**Neurometabolites**						
Asp	3.17	0.57	12.92 ± 3.84	2.80	0.43	14.69 ± 4.19
Cr	4.12	0.49	9.00 ± 3.56	4.14	0.61	9.00 ± 2.71
PCr	6.69	0.85	5.85 ± 2.12	6.45	1.26	6.62 ± 2.29
GABA	1.56	0.29	11.46 ± 1.66	1.62	0.26	11.62 ± 1.61
Gln	3.85	0.71	4.69 ± 0.48	4.06	0.74	4.54 ± 0.52
Glu	13.62	1.44	1.85 ± 0.38	13.47	1.50	1.92 ± 0.28
GPC	1.96	0.27	6.69 ± 4.46	1.87	0.38	6.38 ± 5.47
GSH	1.54	0.21	7.15 ± 1.14	1.53	0.20	7.31 ± 1.18
Ins	8.59	0.73	2.08 ± 0.28	8.31	0.92	2.08 ± 0.28
Lac	0.78	0.18	16.15 ± 3.51	0.92	0.26	14.46 ± 1.90
NAA	12.02	1.27	1.85 ± 0.55	12.39	1.73	1.77 ± 0.44
NAAG	1.48	0.27	8.62 ± 2.53	1.40	0.25	8.92 ± 1.19
Tau	2.31	0.41	8.08 ± 1.32	2.52	0.45	7.46 ± 1.20
PE	3.77	0.52	5.92 ± 0.95	3.75	0.47	6.08 ± 0.86
NAA + NAAG	13.50	1.24	1.69 ± 0.48	13.79	1.86	1.38 ± 0.51
Glx	17.47	2.04	1.85 ± 0.38	17.53	2.16	1.85 ± 0.38
Glx/GABA	11.36	1.25	–	10.95	1.05	–
**Quality control metrics**						
SNR (water suppressed spectra, LCModel output)	45.92	6.64	–	49.28	6.51	–
NAA SNR (water suppressed spectra)	18.88	3.5	–	20.83	2.75	–
FWHM (Hz) (unsuppressed water linewidth)	11.49	1.3	–	11.91	1.64	–
**Percent tissue fraction**						
White matter	0.16	0.1	–	0.11	0.05	–
Gray matter	0.63	0.07	–	0.63	0.04	–
CSF	0.21	0.04	–	0.24	0.07	–

The table presents the mean absolute concentrations of neurometabolites and corresponding Cramér–Rao lower bounds [CRLB (%)] for healthy controls and Crohn’s disease patients. In addition, LCModel-derived spectral quality measures are reported, including the average full width at half maximum (FWHM, in Hz), signal-to-noise ratio (SNR) of the water-suppressed spectra and FWHM of unsuppressed water spectra (averaged across subjects for each group). Additionally, tissue segmentation values (percent tissue fractions) within the MRS voxel in the insula are reported as percentages of gray matter, white matter and cerebrospinal fluid (CSF). Asp, aspartate; Cr, creatine; PCr, phosphocreatine; GABA, gamma-aminobutyric acid; Gln, glutamine; Glu, Glutamate; GPC, glycerylphosphorylcholine; GSH, glutathione; Ins, myo-Inositol; Lac, lactate; NAA, *N*-acetylaspartate; NAAG, *N*-acetylaspartylglutamate; Tau, taurine; PE, phosphoethanolamine; Glx, Glu + Gln; SNR, signal-to-noise ratio; FWHM, full width at half maximum; CSF, cerebrospinal fluid.

### Differences in neurometabolite concentrations between Crohn’s disease patients and healthy controls

**FIGURE 2:**
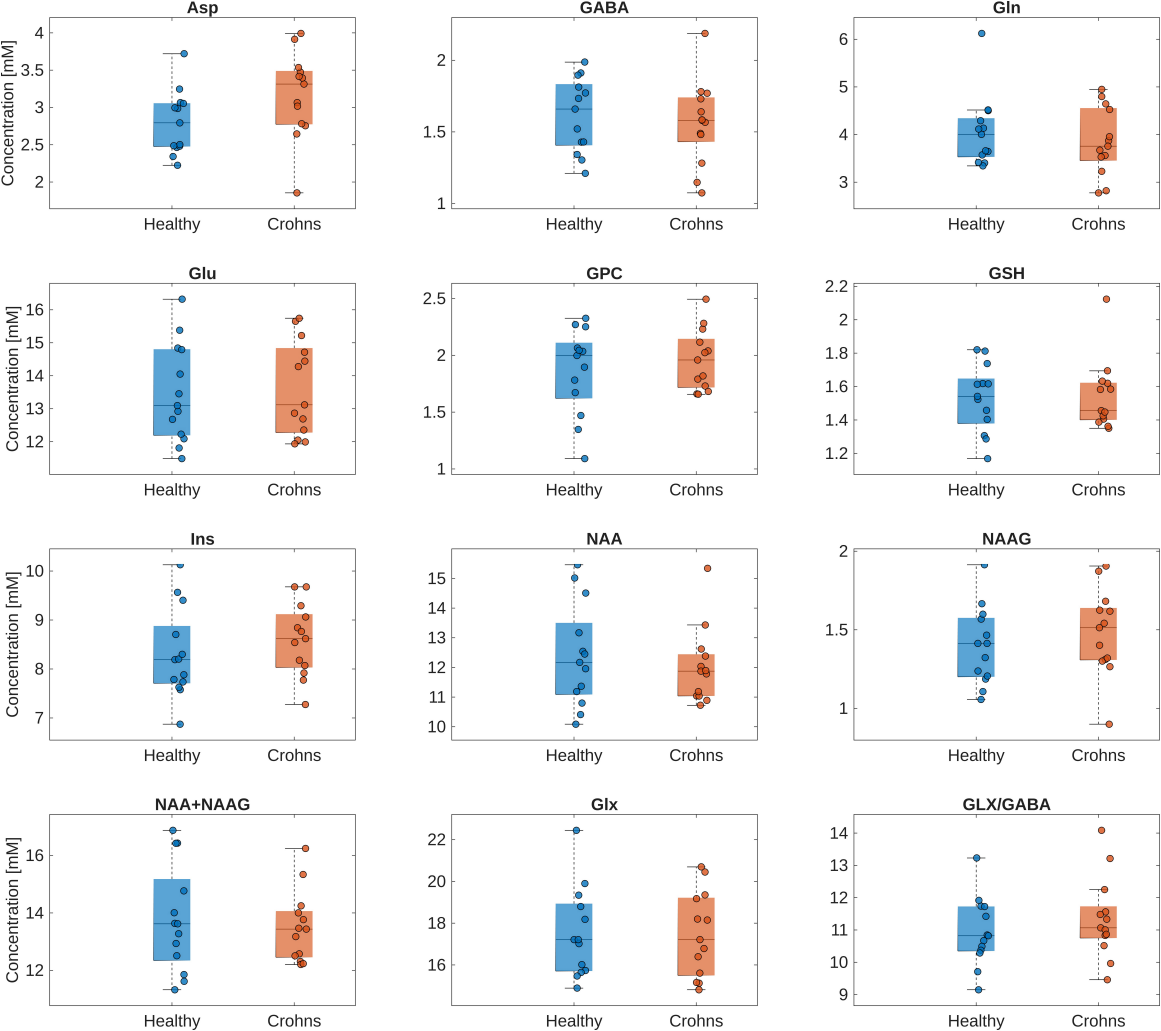
Box plot of the absolute concentrations of neurometabolites in the insula of healthy controls and Crohn’s disease patients. Individual data points are overlaid to illustrate the distribution within each group. The regression analysis showed no significant difference in absolute concentrations of the investigated neurometabolites between the healthy controls and Crohn’s disease patients. Asp, aspartate; GABA, gamma-aminobutyric acid; Glu, glutamate; GPC, glycerylphosphorylcholine; GSH, glutathione; Ins, myo-Inositol; NAA, *N*-acetylaspartate; NAAG, *N*-acetylaspartylglutamate; Glx, Glu + Gln.

No significant differences were found in a regression analysis comparing absolute concentrations of neurometabolites in Crohn’s disease patients and healthy controls (Figure 2 and Supplementary Table 1). Furthermore, even after including medication details as additional binary covariates in the regression analysis, no significant difference was observed.

### Association of Asp, NAAG, and Ins concentrations with gastrointestinal symptoms

**FIGURE 3:**
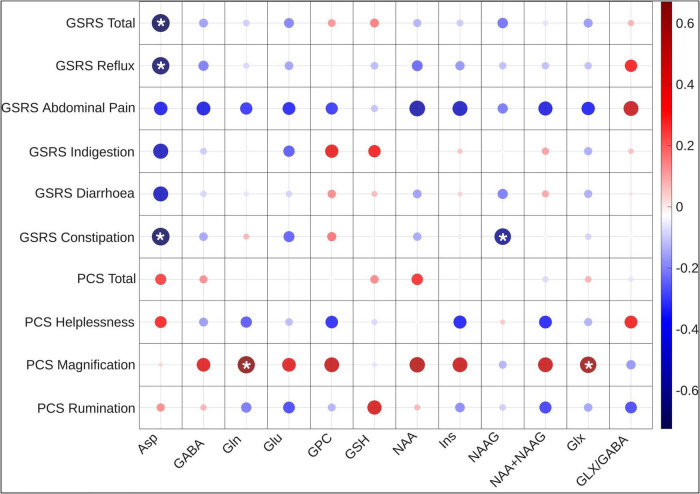
Correlation heatmap of neurometabolite concentrations with PCS and GSRS scores. Significant associations with pain magnification and gastrointestinal symptoms are highlighted with a * sign. This heatmap shows the significant negative correlations of Asp, and Ins with the total GSRS score and subscale scores, as well as positive correlations of Gln, Glx, NAA, NAA + NAAG with PCS pain magnification scores. Asp, aspartate; GABA, gamma-aminobutyric acid; Gln, glutamine; Glu = Glutamate, Glu, glutamate; GPC, glycerylphosphorylcholine; GSH, glutathione; Ins, myo-Inositol; NAA, *N*-acetylaspartate; NAAG, *N*-acetylaspartylglutamate; Glx, Glu + Gln; GSRS, Gastrointestinal Symptom Rating Scale; PCS, Pain Catastrophizing Score.

The Spearman’s correlation analysis of neurometabolite concentrations in Crohn’s disease patients and GSRS scores revealed a significant negative correlation between Asp and the total GSRS score, as well as the subscales for reflux and constipation. Furthermore, a negative significant correlation was revealed between NAAG and the GSRS subscale for constipation. Additionally, a near significant negative correlation was also observed between Ins and the GSRS subscale for abdominal pain (Figure 3). For each comparison, the observed correlation coefficient (ρ), adjusted *p*-value, and critical correlation interval (CI) are reported in Supplementary Table 2.

### Association of Gln, Glx, NAA, and NAA + NAAG concentrations with pain catastrophizing

Following FWER corrections, the Spearman’s correlation coefficients revealed significant associations between the pain magnification score and the absolute values of neurometabolites. Specifically, a positive correlation was observed between pain magnification and the neurometabolites Gln and Glx, as well as the near-significant neurometabolites NAA and NAA + NAAG (Figure 3 and Supplementary Table 3).

## Discussion

This study aimed to investigate the neurometabolic basis of pain perception within the left insula of patients with Crohn’s disease. The primary objectives were to determine (1) whether neurometabolic differences exist in the insula between Crohn’s disease patients and healthy controls and (2) whether these differences are associated with the symptoms experienced by Crohn’s disease patients. To this end, neurometabolites were successfully measured, quantified and subjected to statistical analyses.

In examining the first hypothesis, no significant differences were found in the absolute concentrations of neurometabolites between Crohn’s disease patients and healthy controls. This finding may stem from the study’s design, as MRS data were acquired during the brain’s resting state and not during the active application of a painful stimulus. Prior studies have shown that neurometabolite concentrations tend to show more drastic changes when assessed during active stimulus application compared to the resting state in chronic pain conditions (Gutzeit et al., 2011, 2013; Harfeldt et al., 2018). It is reasonable to assume that this is why the Crohn’s disease patients measured in our study, who were not in acute pain during the MRS measurement, did not show a significant difference in absolute metabolite concentrations compared to healthy controls. Furthermore, most of the patients included in the study were receiving treatment for Crohn’s disease, which may have influenced the results. For instance, a study by Kong et al. (2022) indicated that heightened Glu concentrations in the brain are only observed in patients during acute stages of the disease, suggesting that treatment or disease remission might mitigate these changes.

Regarding the second hypothesis, the findings suggest that alterations in insula neurometabolites in Crohn’s disease patients may be associated with the manifestation of symptoms and pain catastrophizing. Specifically, a negative correlation was detected between GSRS scores and the neurometabolites Asp and NAAG, as well as a near significant correlation with Ins. Furthermore, Gln and Glx, as well as the near significant NAA and NAA + NAAG, were positively correlated with the PCS subscale of pain magnification.

These findings are consistent with current understanding relating to the role of the insula in interoception and, therefore, pain processing. The insula is notably implicated in gut-related interoceptive processes, as demonstrated by correlations between the sensory reaction of the gastrointestinal tract and increased blood oxygenation level dependent (BOLD) signals in the insula (Larsson et al., 2012). With regard to pain processing, as mentioned previously, the posterior insula serves as a processing site for the somatosensory aspects of pain, while the anterior insula processes the emotional aspects of pain (Craig, 2002). Moreover, it has also been proposed that the insula assumes a distinct function in processing visceral pain (Dunckley et al., 2005), implying that it plays an important role in the pain processing of Crohn’s disease patients. Another study has shown that insula activity and pain sensitivity increase during systemic inflammation, a hallmark of Crohn’s disease (Karshikoff et al., 2016), further indicating the role of the insula in increased pain perception during inflammation. Generally, increased pain intensity was shown to be positively correlated with higher insula activity (Mathur et al., 2016), making these findings particularly relevant.

The negative correlation between GSRS scores and Asp and NAAG and similar trend observed in Ins, can be interpreted as follows: Aspartate functions as an excitatory neurotransmitter that engages the *N*-methyl-D-aspartate receptor (NMDAR), which is implicated in the modulation of visceral pain hypersensitivity and, consequently, in central sensitization to pain (Gaudreau and Plourde, 2004; Willert et al., 2004). Our results show a negative correlation between Asp levels and the manifestation of Crohn’s disease symptoms, a paradox which could be explained by neuroprotective mechanisms downregulating Asp levels to protect the brain from excitotoxicity. Given that Asp activates the NMDAR, reduced Asp levels may produce symptoms resembling those of anti-NMDAR encephalitis. Supporting this, several studies have reported that treatment with TNF-α-inhibitors in Crohn’s disease patients could potentially lead to the onset of anti-NMDAR encephalitis (MacKay and Salh, 2024; Noble and Lancaster, 2018; Oh et al., 2022). Several of the patients in this study had indeed received treatment with TNF-α-inhibitors. Furthermore, studies have demonstrated that individuals with anti-NMDAR encephalitis exhibit disrupted functional connectivity (FC) and decreased coupling between cerebral blood flow (CBF) and regional homogeneity (ReHo) (CBF-ReHo coupling) in the insula (Guo et al., 2022; Li et al., 2021). These alterations in CBF-ReHo coupling were associated with deficits in executive functioning (Guo et al., 2022). A study by Abeare et al. (2010) demonstrated a negative correlation between executive functioning and pain levels in patients with rheumatoid arthritis experiencing chronic pain, indicating that a decrease in executive functioning is related to elevated pain levels. The disrupted functional connectivity may also contribute to increased pain, as observed in complex regional pain syndrome (CRPS), a comparable chronic pain condition (Kim et al., 2023). This approach may provide an explanation for the noted negative correlation between Asp levels and GSRS scores in our study. However, this remains exploratory and warrants further research.

N-acetylaspartylglutamate (NAAG) functions as a neurotransmitter that modulates glutamatergic signaling primarily through the inhibition of glutamate release. NAAG is hydrolyzed by the enzyme glutamate carboxypeptidase II (GCPII), which converts it into NAA and glutamate. Increased GCPII activity has been reported within affected intestinal regions of patients with IBD and is thought to contribute to disease symptoms (Rais et al., 2016). We hypothesize that the observed negative correlation between NAAG levels and gastrointestinal symptom severity may result from this increased GCPII activity, extending to the brain and specifically the insula, resulting in a decrease in NAAG levels and a consecutive increase in glutamate levels. This imbalance could lead to excitotoxicity and exacerbate nociceptive signaling, given the proposed role of NAAG in modulating pain perception (Adedoyin et al., 2010). This is further supported by the fact that inhibitors of GCPII were shown to elevate NAAG levels and decrease glutamate levels, thereby reducing pain and glutamate-mediated excitotoxicity (Adedoyin et al., 2010; Slusher et al., 1999; Wozniak et al., 2012).

Myo-Inositol serves as a well-established glial marker due to its preferential uptake by astrocytes and its role in maintaining cerebral osmotic homeostasis, while also being associated with microglial activity (Ebert et al., 2021; Isaacks et al., 1994). Emerging evidence also suggests a role for myo-inositol in supporting GABAergic signaling, including modulation of GABA-A receptor subunits and participation in inositol phosphate pathways relevant to GABA-B receptor function (Solomonia et al., 2013). Therefore, the observed reduction in Ins levels may not only reflect glial dysfunction, a loss in glial density or altered glutamatergic signaling but could also suggest a relative reduction in inhibitory GABAergic tone. This dual contribution may enhance excitability within the insular cortex, which could be particularly relevant in the context of chronic pain and interoceptive dysregulation. This interpretation is further supported by previous findings showing decreased myo-inositol in the insula during acute dental pain volunteers (Gutzeit et al., 2011, 2013).

The results of this study also indicate that elevated Gln and Glx levels, as well as similar trend of elevated NAA and NAA + NAAG levels in Crohn’s disease patients could be associated with increased pain magnification scores on the PCS. Pain catastrophizing involves three domains: first, the inability to divert one’s attention from thoughts associated with pain, i.e., rumination; second, the amplification of perceived pain, i.e., magnification; and third, helplessness when confronted with painful circumstances (Sullivan et al., 1995). It has been demonstrated that people engaging in pain catastrophizing have difficulty moving their focus away from pain and tend to overvalue painful stimuli, causing an increase in pain severity through pain hypervigilance (Goubert et al., 2004; Sullivan et al., 2001; Van Damme et al., 2004).

Consequently, when viewed in terms of our findings, we hypothesize that the elevated levels of neurometabolites may reflect an increase in insula activity and interoceptive awareness, which, in turn, causes patients to focus more on their pain. This could then lead to pain amplification, resulting in an increase in pain magnification. This aligns with evidence suggesting that increased insular cortex activation is associated with elevated levels of pain catastrophizing (Mathur et al., 2016; Seminowicz and Davis, 2006).

The connection between our findings and interoceptive awareness can be explored further: studies have demonstrated a positive correlation between Glu levels in the insula and interoceptive awareness (Ernst et al., 2013; Wiebking et al., 2014). Glutamate is an excitatory neurotransmitter (Bennett and Balcar, 1999) involved in interoception and pain processing in the insula. Consequently, elevated concentrations of Glx, alongside Gln, which is intrinsically involved in Glu metabolism and strongly correlated with Glu levels (Rae, 2014), might reflect higher insula activity. This, in turn, likely contributes to higher interoceptive awareness and pain sensitivity. Such changes could lead to persistent pain awareness and magnification, as pain hypervigilance leads to an increase in pain severity as mentioned above (Goubert et al., 2004).

Supporting this, Ernst et al. also proposed that elevated Glu levels, and therefore higher insula activation, may lead to the insula misassigning salience to stimuli of little importance (Ernst et al., 2013). As part of the salience network (SN) (Seeley et al., 2007), the insula plays a role in directing attention to salient stimuli by modulating the activity of the executive control network (ECN) and the default mode network (DMN) (Goulden et al., 2014; Menon and Uddin, 2010) by acting as a junction between internal and external salient information (Uddin, 2015). The idea that the insula wrongly assigns salience to painful stimuli, contributing to heightened pain perception through increased insula activity in Crohn’s disease, is further supported by the fact that increased functional connectivity (FC) between the SN and the ECN has also been observed in IBD patients (Kornelsen et al., 2020), suggesting that IBD patients might also experience heightened sensitivity to salient stimuli (Kornelsen et al., 2020). Furthermore, a reduction in FC was observed within the DMN, suggesting that individuals with Crohn’s disease may exhibit a diminished capacity to redirect their focus away from pain (Kornelsen et al., 2020). This is additionally supported by a study by Mathur et al. on chronic pain in migraine patients, which demonstrated that inactivity in critical regions of the DMN resulted in increased pain catastrophizing (Mathur et al., 2016), further validating the supposition that DMN dysfunction causes an increased focus on pain.

These findings suggest that the elevated levels of Glx and Gln observed in our study could reflect increased insula activity in Crohn’s disease patients. This heightened insula activity may contribute to greater interoceptive awareness and heightened sensitivity to pain. Additionally, the insula’s role in evaluating the salience and significance of pain perception could result in a disproportionate focus on pain, ultimately leading to pain magnification in Crohn’s disease patients. Moreover, the observed trend of positive association between levels of NAA and NAA + NAAG and pain magnification further underscores the link between heightened neural activity and insula activation. That is to say, as NAA is known to be an indicator of neuronal health (Rae, 2014; Tsai and Coyle, 1995), the observed increase may reflect higher neuronal viability and, therefore, possibly higher insular activity, further supporting the aforementioned theory. A study by Lee et al. presented similar results based on fibromyalgia, another condition characterized by chronic pain. They found that in fibromyalgia patients, Glx and NAA concentrations in the insula were positively correlated with PCS scores (Lee et al., 2021), suggesting that our findings are not exclusive to Crohn’s disease but are applicable to other chronic pain conditions.

This study presents several limitations. The relatively small sample size may constrain the generalizability of the findings; thus, future research should aim to include larger cohorts to enhance statistical power and external validity. Although water-referenced quantification avoids reliance on metabolite ratios (e.g., Cr-normalization), it is not without limitations. Variability in tissue voxel composition (Gogishvili et al., 2023), brain water content and relaxation properties—particularly in pathological conditions—can affect reference signal stability and should be considered when interpreting absolute concentration values. Additionally, incorporating comprehensive gut microbiota profiling could elucidate the influence of microbial composition on neurometabolite concentrations, given the established role of the gut-brain axis in modulating neurochemical pathways. Focusing exclusively on treatment-naïve patients during the active phase of Crohn’s disease may also yield novel insights into disease-specific neurobiological alterations. Alternatively, future investigations that include patients receiving ongoing treatment should organize participants into subgroups based on the specific therapies received.

This study demonstrates that gastrointestinal symptoms associated with Crohn’s disease may be partially linked to altered neurometabolite levels in the insular cortex. These alterations may contribute to increased insular activity, leading to heightened interoceptive awareness and pain sensitivity, which in turn may exacerbate pain catastrophizing. These findings provide deeper insights into the critical role of the insula in inflammatory and chronic pain conditions.

## Data Availability

The raw data supporting the conclusions of this article will be made available by the authors, without undue reservation.

## Footnotes

1
https://mrshub.org/datasets_mm

## Supplementary material

Supplementary Figure 1 Swarm plots showing the distribution of absolute Cramér-Rao Lower Bound (aCRLB) values across metabolites in healthy controls (HC) and Crohn’s disease (CD) patients. Individual data points are shown as jittered scatter dots for each group. The solid horizontal lines indicate the group mean, and vertical lines represent ± 2 standard deviations (SD) around the mean. Asp, aspartate; GABA, gamma-aminobutyric acid; Gln, glutamine; Glu = Glutamate, Glu, glutamate; GPC, glycerylphosphorylcholine; GSH, glutathione; Ins, myo-Inositol; NAA, *N*-acetylaspartate; NAAG, *N*-acetylaspartylglutamate; Glx, Glu + Gln.

Supplementary Figure 2 Swarm plots showing spectral quality metrics across participants. **(Left)** NAA-based SNR computed from individual transients; **(Right)** LCModel-derived SNR from the averaged spectrum of each subject. Individual data points are shown as jittered scatter dots for each group [healthy controls (HC) and Crohn’s disease (CD) patients]. Solid horizontal lines indicate the group mean, and vertical lines represent ± 2 standard deviations (SD) used as the threshold for identifying low-quality spectra.
