# Supplementary material for: Neurometabolic signatures of gastrointestinal symptoms in the insula of Crohn’s disease patients: explorative findings from a 7T MRS study
Source: Front Hum Neurosci. 2025 Nov 20;19:1620488. doi: 10.3389/fnhum.2025.1620488 (PMC12677143; doi:10.3389/fnhum.2025.1620488)
Supplement: Supplementary file 1 [file Table_1.docx]

| **Metabolite** | **Variable** | **Coefficient Estimate** | **Standard Error** | **t-Statistic** | **p-Value** | **Root Mean Squared Error** | **Adjusted R-Squared** | **F-statistic vs. constant model** | **p-value** |
| --- | --- | --- | --- | --- | --- | --- | --- | --- | --- |
| Asp | Intercept | 17.09 | 7.292 | 2.3436 | 0.028541 | 7.32 | 0.0849 | 1.77 | 0.182 |
|  | Group (Healthy vs. Crohn) | 6.3392 | 2.9219 | 2.1696 | 0.041116 |  |  |  |  |
|  | Gender | -1.8494 | 3.137 | -0.58956 | 0.56149 |  |  |  |  |
|  | Age | -0.20035 | 0.26437 | -0.75786 | 0.45658 |  |  |  |  |
| GABA | Intercept | 17.319 | 7.1719 | 2.4149 | 0.024498 | 7.2 | 0.115 | 2.08 | 0.132 |
|  | Group (Healthy vs. Crohn) | -1.3169 | 2.8737 | -0.45827 | 0.65125 |  |  |  |  |
|  | Gender | 6.9621 | 3.0853 | 2.2565 | 0.034311 |  |  |  |  |
|  | Age | -0.29184 | 0.26001 | -1.1224 | 0.27378 |  |  |  |  |
| Gln | Intercept | 14.987 | 7.0172 | 2.1358 | 0.044074 | 7.04 | 0.153 | 2.5 | 0.086 |
|  | Group (Healthy vs. Crohn) | -0.48617 | 2.8117 | -0.17291 | 0.86431 |  |  |  |  |
|  | Gender | 7.9751 | 3.0188 | 2.6418 | 0.014869 |  |  |  |  |
|  | Age | -0.2474 | 0.2544 | -0.97248 | 0.34138 |  |  |  |  |
| Glu | Intercept | 1.738 | 7.9185 | 2.2401 | 0.035514 | 7.95 | -0.0791 | 0.389 | 0.762 |
|  | Group (Healthy vs. Crohn) | 1.1935 | 3.1729 | 0.37616 | 0.7104 |  |  |  |  |
|  | Gender | 2.5488 | 3.4065 | 0.7482 | 0.46226 |  |  |  |  |
|  | Age | -0.24133 | 0.28708 | -0.84062 | 0.4096 |  |  |  |  |
| GPC | Intercept | -1.1285 | 6.5342 | -0.1727 | 0.86446 | 6.56 | 0.265 | 4.01 | 0.0204 |
|  | Group (Healthy vs. Crohn) | 0.18049 | 2.6182 | 0.068937 | 0.94566 |  |  |  |  |
|  | Gender | 8.3398 | 2.811 | 2.9668 | 0.0071207 |  |  |  |  |
|  | Age | 0.3205 | 0.23689 | 1.353 | 0.18981 |  |  |  |  |
| GSH | Intercept | 15.017 | 8.098 | 1.8544 | 0.077141 | 8.13 | -0.129 | 0.0508 | 0.984 |
|  | Group (Healthy vs. Crohn) | -0.22403 | 3.2448 | -0.069042 | 0.94558 |  |  |  |  |
|  | Gender | 1.0252 | 3.4837 | 0.29428 | 0.7713 |  |  |  |  |
|  | Age | -0.07732 | 0.29359 | -0.26337 | 0.79472 |  |  |  |  |
| Ins | Intercept | 7.3033 | 6.8766 | 1.0621 | 0.29973 | 6.9 | 0.186 | 2.91 | 0.0574 |
|  | Group (Healthy vs. Crohn) | 3.2264 | 2.7554 | 1.1709 | 0.25416 |  |  |  |  |
|  | Gender | 7.9997 | 2.9583 | 2.7042 | 0.012957 |  |  |  |  |
|  | Age | -0.034915 | 0.24931 | -0.14005 | 0.8899 |  |  |  |  |
| NAA | Intercept | 6.7592 | 7.4209 | 0.91083 | 0.37226 | 7.45 | 0.0523 | 1.46 | 0.253 |
|  | Group (Healthy vs. Crohn) | -2.0473 | 2.9735 | -0.68852 | 0.49832 |  |  |  |  |
|  | Gender | 5.9269 | 3.1924 | 1.8565 | 0.076818 |  |  |  |  |
|  | Age | 0.13389 | 0.26904 | 0.49765 | 0.62367 |  |  |  |  |
| NAAG | Intercept | -3.4176 | 7.2034 | -0.47445 | 0.63986 | 7.23 | 0.107 | 2.0 | 0.144 |
|  | Group (Healthy vs. Crohn) | 1.5022 | 2.8863 | 0.52045 | 0.60795 |  |  |  |  |
|  | Gender | 0.71773 | 3.0989 | 0.23161 | 0.81898 |  |  |  |  |
|  | Age | 0.57301 | 0.26115 | 2.1942 | 0.039078 |  |  |  |  |
| NAA+NAAG | Intercept | 2.7524 | 7.2592 | 0.37917 | 0.7082 | 7.28 | 0.0931 | 1.86 | 0.167 |
|  | Group (Healthy vs. Crohn) | -1.5559 | 2.9087 | -0.5349 | 0.59808 |  |  |  |  |
|  | Gender | 6.0762 | 3.1229 | 1.9457 | 0.064576 |  |  |  |  |
|  | Age | 0.26764 | 0.26318 | 1.017 | 0.32023 |  |  |  |  |
| Glx | Intercept | 18.67 | 7.5298 | 2.4794 | 0.021302 | 7.56 | 0.0243 | 1.21 | 0.33 |
|  | Group (Healthy vs. Crohn) | 0.61351 | 3.0171 | 0.20334 | 0.84074 |  |  |  |  |
|  | Gender | 5.2208 | 3.2393 | 1.6117 | 0.12128 |  |  |  |  |
|  | Age | -0.33243 | 0.27298 | -1.2178 | 0.23622 |  |  |  |  |
| GLX/GABA | Intercept | 10.515 | 7.014 | 1.4991 | 0.14806 | 7.04 | 0.153 | 2.51 | 0.0852 |
|  | Group (Healthy vs. Crohn) | 2.1557 | 2.8105 | 0.76702 | 0.45122 |  |  |  |  |
|  | Gender | -7.4508 | 3.0174 | -2.4693 | 0.021778 |  |  |  |  |
|  | Age | 0.25837 | 0.25429 | 1.0161 | 0.32064 |  |  |  |  |
