# Supplementary material for: Neurometabolic signatures of gastrointestinal symptoms in the insula of Crohn’s disease patients: explorative findings from a 7T MRS study
Source: Front Hum Neurosci. 2025 Nov 20;19:1620488. doi: 10.3389/fnhum.2025.1620488 (PMC12677143; doi:10.3389/fnhum.2025.1620488)
Supplement: Supplementary file 2 [file Table_2.docx]

| Metabolite | Pain Score | Rho | Corr Lower CI | Corr Upper CI | Estimated Alpha | p-Value |
| --- | --- | --- | --- | --- | --- | --- |
| Asp | \| GSRS Total \| \| --- \| | -0.725 | -0.5709 | 0.599 | 0.0514 | 0.0082 |
|  | Reflux | -0.6784 | -0.5848 | 0.5848 | 0.052 | 0.0198 |
|  | Abdominal Pain | -0.4149 | -0.5869 | 0.5745 | 0.0501 | 0.1742 |
|  | Indigestion | -0.5141 | -0.5775 | 0.5775 | 0.0511 | 0.0886 |
|  | Diarrhea | -0.5088 | -0.5746 | 0.5765 | 0.0506 | 0.0892 |
|  | Constipation | -0.7076 | -0.5927 | 0.5765 | 0.0505 | 0.0138 |
| GABA | \| GSRS Total \| \| --- \| | -0.1786 | -0.5779 | 0.5779 | 0.0523 | 0.5656 |
|  | Reflux | -0.2339 | -0.5848 | 0.5887 | 0.0513 | 0.4522 |
|  | Abdominal Pain | -0.4327 | -0.5923 | 0.5869 | 0.0502 | 0.1604 |
|  | Indigestion | -0.1056 | -0.5986 | 0.5775 | 0.0508 | 0.7428 |
|  | Diarrhea | -0.0915 | -0.5911 | 0.5893 | 0.0503 | 0.7598 |
|  | Constipation | -0.1688 | -0.5747 | 0.5927 | 0.0511 | 0.5898 |
| Gln | \| GSRS Total \| \| --- \| | -0.1016 | -0.5849 | 0.5709 | 0.0505 | 0.7546 |
|  | Reflux | -0.0858 | -0.5771 | 0.5926 | 0.0515 | 0.782 |
|  | Abdominal Pain | -0.3405 | -0.5816 | 0.5816 | 0.0511 | 0.2634 |
|  | Indigestion | -0.007 | -0.5916 | 0.5845 | 0.0522 | 0.9634 |
|  | Diarrhea | -0.0622 | -0.571 | 0.5838 | 0.0504 | 0.836 |
|  | Constipation | 0.0826 | -0.5819 | 0.5891 | 0.0506 | 0.7936 |
| Glu | \| GSRS Total \| \| --- \| | -0.2207 | -0.5779 | 0.5814 | 0.0504 | 0.4686 |
|  | Reflux | -0.1716 | -0.5693 | 0.5771 | 0.051 | 0.5716 |
|  | Abdominal Pain | -0.3688 | -0.5834 | 0.5923 | 0.0511 | 0.2228 |
|  | Indigestion | -0.2887 | -0.5916 | 0.5775 | 0.0514 | 0.3466 |
|  | Diarrhea | -0.0952 | -0.5893 | 0.56 | 0.0507 | 0.7626 |
|  | Constipation | -0.273 | -0.5711 | 0.5819 | 0.0515 | 0.3746 |
| GPC | \| GSRS Total \| \| --- \| | 0.1366 | -0.5884 | 0.5814 | 0.0507 | 0.6686 |
|  | Reflux | -0.0078 | -0.5926 | 0.5848 | 0.0517 | 0.998 |
|  | Abdominal Pain | -0.3334 | -0.5852 | 0.5887 | 0.0507 | 0.287 |
|  | Indigestion | 0.3944 | -0.5845 | 0.5775 | 0.0524 | 0.1946 |
|  | Diarrhea | 0.1574 | -0.5746 | 0.5929 | 0.0509 | 0.6188 |
|  | Constipation | 0.1904 | -0.5747 | 0.5819 | 0.0503 | 0.5658 |
| GSH | \| GSRS Total \| \| --- \| | 0.1786 | -0.5832 | 0.5779 | 0.0503 | 0.5684 |
|  | Reflux | -0.1326 | -0.5771 | 0.5771 | 0.0523 | 0.654 |
|  | Abdominal Pain | -0.117 | -0.5745 | 0.5887 | 0.051 | 0.6918 |
|  | Indigestion | 0.3451 | -0.5845 | 0.5775 | 0.0504 | 0.2608 |
|  | Diarrhea | 0.0805 | -0.582 | 0.582 | 0.0503 | 0.7792 |
|  | Constipation | -0.0144 | -0.5963 | 0.5783 | 0.0509 | 0.993 |
| Ins | \| GSRS Total \| \| --- \| | -0.1471 | -0.5867 | 0.5779 | 0.0501 | 0.6558 |
|  | Reflux | -0.2651 | -0.5771 | 0.5771 | 0.0516 | 0.3838 |
|  | Abdominal Pain | -0.5568 | -0.5887 | 0.5887 | 0.0509 | 0.0666 |
|  | Indigestion | -0.0282 | -0.5845 | 0.5845 | 0.0507 | 0.9064 |
|  | Diarrhea | -0.1757 | -0.5783 | 0.5783 | 0.0505 | 0.5772 |
|  | Constipation | -0.1616 | -0.5711 | 0.5783 | 0.0507 | 0.6168 |
| NAA | \| GSRS Total \| \| --- \| | -0.1086 | -0.5814 | 0.5954 | 0.0504 | 0.725 |
|  | Reflux | -0.1949 | -0.5848 | 0.5848 | 0.0506 | 0.5378 |
|  | Abdominal Pain | -0.5036 | -0.5852 | 0.5816 | 0.0509 | 0.094 |
|  | Indigestion | 0.0634 | -0.5775 | 0.5704 | 0.0531 | 0.8298 |
|  | Diarrhea | 0.0476 | -0.5783 | 0.5673 | 0.0515 | 0.8886 |
|  | Constipation | -0.0503 | -0.5855 | 0.5783 | 0.0513 | 0.8628 |
| NAAG | \| GSRS Total \| \| --- \| | -0.2487 | -0.5849 | 0.5814 | 0.0513 | 0.4256 |
|  | Reflux | -0.1248 | -0.5926 | 0.5771 | 0.0533 | 0.682 |
|  | Abdominal Pain | -0.2376 | -0.5745 | 0.5852 | 0.0509 | 0.4474 |
|  | Indigestion | -0.0282 | -0.574 | 0.5916 | 0.0508 | 0.9196 |
|  | Diarrhea | -0.2342 | -0.582 | 0.5893 | 0.051 | 0.4468 |
|  | Constipation | -0.6178 | -0.5891 | 0.5783 | 0.0515 | 0.0356 |
| NAA + NAAG | \| GSRS Total \| \| --- \| | -0.0701 | -0.5814 | 0.5779 | 0.0508 | 0.8268 |
|  | Reflux | -0.1248 | -0.5693 | 0.5771 | 0.0523 | 0.6864 |
|  | Abdominal Pain | -0.4504 | -0.5781 | 0.5887 | 0.0504 | 0.1336 |
|  | Indigestion | 0.1197 | -0.5704 | 0.5916 | 0.0526 | 0.707 |
|  | Diarrhea | 0.1135 | -0.5966 | 0.582 | 0.0513 | 0.7058 |
|  | Constipation | -0.0323 | -0.5783 | 0.5783 | 0.0518 | 0.9324 |
| Glx | \| GSRS Total \| \| --- \| | -0.1856 | -0.5779 | 0.5954 | 0.0508 | 0.5538 |
|  | Reflux | -0.1248 | -0.5848 | 0.5771 | 0.0537 | 0.6956 |
|  | Abdominal Pain | -0.4007 | -0.5834 | 0.594 | 0.05 | 0.1958 |
|  | Indigestion | -0.1549 | -0.581 | 0.5704 | 0.0513 | 0.625 |
|  | Diarrhea | -0.1537 | -0.582 | 0.582 | 0.0515 | 0.6268 |
|  | Constipation | -0.097 | -0.5891 | 0.5747 | 0.0505 | 0.774 |
| Glx/GABA | \| GSRS Total \| \| --- \| | 0.0876 | -0.5779 | 0.5779 | 0.0503 | 0.7726 |
|  | Reflux | 0.3431 | -0.5771 | 0.5848 | 0.0523 | 0.2566 |
|  | Abdominal Pain | 0.5001 | -0.5816 | 0.5745 | 0.0515 | 0.094 |
|  | Indigestion | 0.0704 | -0.5634 | 0.5916 | 0.0511 | 0.8226 |
|  | Diarrhea | 0.022 | -0.5856 | 0.5856 | 0.051 | 0.9632 |
|  | Constipation | -0.0287 | -0.5855 | 0.5927 | 0.0506 | 0.9284 |

| Metabolite | Pain Score | Rho | Corr Lower CI | Corr Upper CI | Estimated Alpha | p-Value |
| --- | --- | --- | --- | --- | --- | --- |
| Asp | PCS Total | 0.2759 | -0.6115 | 0.6069 | 0.0504 | 0.4132 |
|  | Helplessness | 0.3141 | -0.6144 | 0.619 | 0.0514 | 0.363 |
|  | Magnification | 0.0372 | -0.6141 | 0.6071 | 0.0506 | 0.9104 |
|  | Rumination | 0.1521 | -0.5991 | 0.6084 | 0.0511 | 0.6444 |
| GABA | PCS Total | 0.1471 | -0.6023 | 0.6069 | 0.0518 | 0.6582 |
|  | Helplessness | -0.1802 | -0.6144 | 0.6098 | 0.0507 | 0.5742 |
|  | Magnification | 0.4187 | -0.6095 | 0.6048 | 0.0515 | 0.1998 |
|  | Rumination | 0.0876 | -0.6176 | 0.6037 | 0.0508 | 0.782 |
| Gln | PCS Total | -0.0046 | -0.6161 | 0.6046 | 0.0501 | 0.9584 |
|  | Helplessness | -0.2864 | -0.619 | 0.6052 | 0.0511 | 0.3744 |
|  | Magnification | 0.6699 | -0.6048 | 0.6095 | 0.0516 | 0.0302 |
|  | Rumination | -0.2397 | -0.6084 | 0.6037 | 0.051 | 0.4524 |
| Glu | PCS Total | 0.0138 | -0.6092 | 0.6161 | 0.0504 | 0.97 |
|  | Helplessness | -0.134 | -0.6052 | 0.6098 | 0.0508 | 0.6628 |
|  | Magnification | 0.4187 | -0.6095 | 0.6025 | 0.0505 | 0.182 |
|  | Rumination | -0.3134 | -0.6084 | 0.6037 | 0.0528 | 0.3302 |
| GPC | PCS Total | -0.023 | -0.6069 | 0.6161 | 0.0518 | 0.9314 |
|  | Helplessness | -0.3557 | -0.6052 | 0.5982 | 0.0505 | 0.2768 |
|  | Magnification | 0.4932 | -0.6188 | 0.6025 | 0.0503 | 0.119 |
|  | Rumination | -0.1429 | -0.6084 | 0.6268 | 0.0506 | 0.6608 |
| GSH | PCS Total | 0.1609 | -0.6023 | 0.6115 | 0.051 | 0.6328 |
|  | Helplessness | -0.0832 | -0.619 | 0.6005 | 0.0514 | 0.798 |
|  | Magnification | -0.0605 | -0.6048 | 0.6095 | 0.051 | 0.8616 |
|  | Rumination | 0.4563 | -0.6037 | 0.613 | 0.0512 | 0.1596 |
| Ins | PCS Total | 0.2943 | -0.6069 | 0.6069 | 0.0516 | 0.365 |
|  | Helplessness | -0.0323 | -0.6098 | 0.6052 | 0.0506 | 0.9172 |
|  | Magnification | 0.535 | -0.6048 | 0.6002 | 0.0515 | 0.0882 |
|  | Rumination | 0.083 | -0.6107 | 0.6084 | 0.0514 | 0.8126 |
| NAA | PCS Total | -0.0414 | -0.6 | 0.5931 | 0.0505 | 0.9022 |
|  | Helplessness | -0.3834 | -0.6098 | 0.6052 | 0.0511 | 0.2306 |
|  | Magnification | 0.4932 | -0.6048 | 0.6095 | 0.0509 | 0.1192 |
|  | Rumination | -0.212 | -0.6084 | 0.6176 | 0.0509 | 0.5214 |
| NAAG | PCS Total | -0.0184 | -0.6069 | 0.6069 | 0.0517 | 0.967 |
|  | Helplessness | 0.0554 | -0.6098 | 0.6005 | 0.0509 | 0.8424 |
|  | Magnification | -0.1442 | -0.6095 | 0.6095 | 0.0511 | 0.6536 |
|  | Rumination | -0.1014 | -0.6084 | 0.6153 | 0.0506 | 0.7666 |
| NAA + NAAG | PCS Total | -0.0828 | -0.6023 | 0.6046 | 0.0514 | 0.8022 |
|  | Helplessness | -0.3696 | -0.6029 | 0.6098 | 0.0502 | 0.2552 |
|  | Magnification | 0.4932 | -0.6141 | 0.6188 | 0.051 | 0.1222 |
|  | Rumination | -0.3272 | -0.6084 | 0.5899 | 0.0511 | 0.3192 |
| Glx | PCS Total | 0.0966 | -0.5977 | 0.6115 | 0.0509 | 0.7866 |
|  | Helplessness | -0.1478 | -0.6052 | 0.5959 | 0.0526 | 0.6534 |
|  | Magnification | 0.6048 | -0.6095 | 0.6095 | 0.0502 | 0.0502 |
|  | Rumination | -0.1659 | -0.5991 | 0.6084 | 0.051 | 0.627 |
| Glx/GABA | PCS Total | -0.0644 | -0.6207 | 0.6069 | 0.0508 | 0.8392 |
|  | Helplessness | 0.3696 | -0.6144 | 0.6005 | 0.0506 | 0.248 |
|  | Magnification | -0.1954 | -0.6002 | 0.6095 | 0.0528 | 0.5474 |
|  | Rumination | -0.3134 | -0.5853 | 0.5945 | 0.0519 | 0.3274 |
