# Supplementary figures and images for: Neurometabolic signatures of gastrointestinal symptoms in the insula of Crohn’s disease patients: explorative findings from a 7T MRS study
Source: Front Hum Neurosci. 2025 Nov 20;19:1620488. doi: 10.3389/fnhum.2025.1620488 (PMC12677143; doi:10.3389/fnhum.2025.1620488)

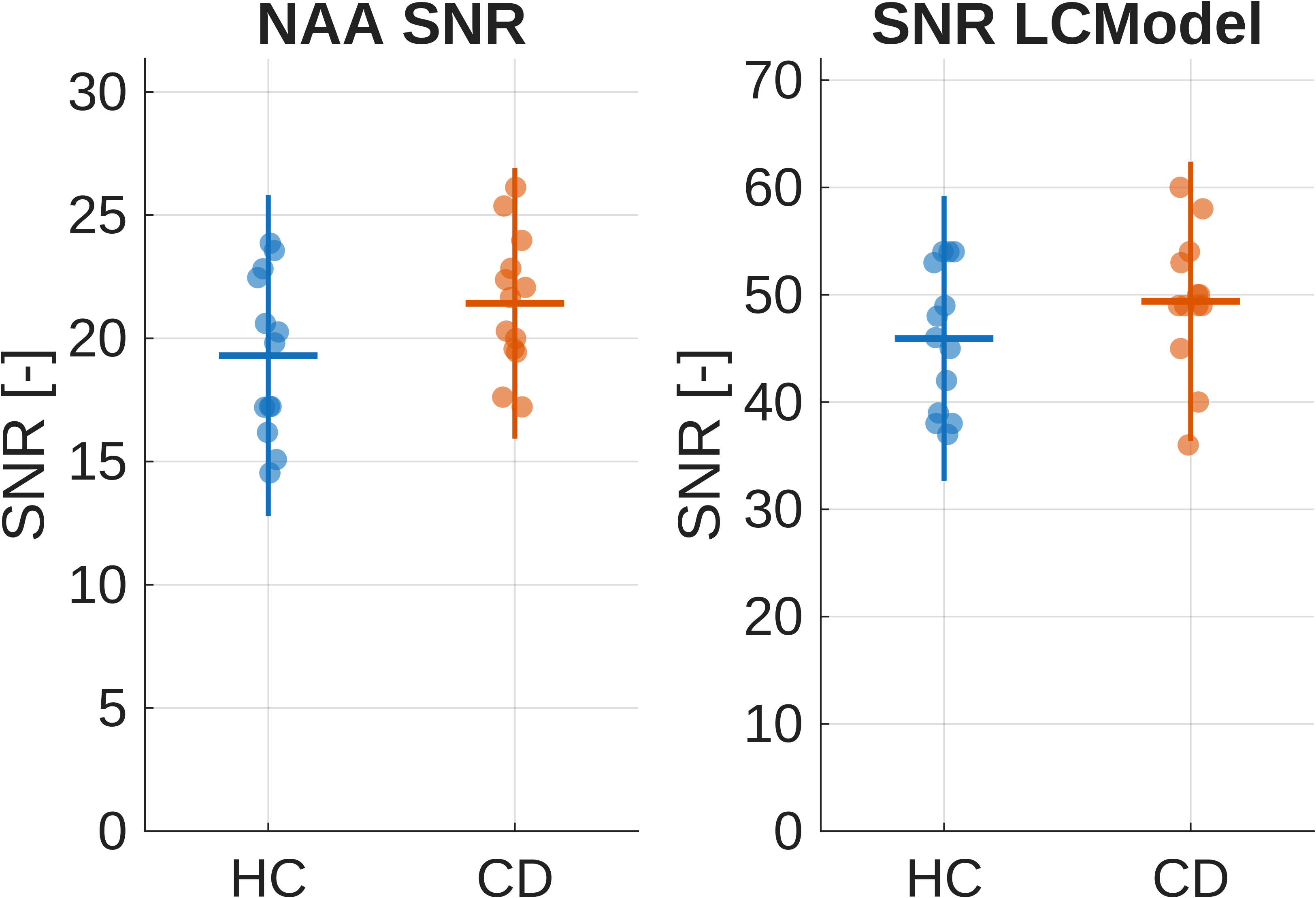

Supplement: Supplementary Figure 2 — Swarm plots showing spectral quality metrics across participants. (Left) NAA-based SNR computed from individual transients; (Right) LCModel-derived SNR from the averaged spectrum of each subject. Individual data points are shown as jittered scatter dots for each group [healthy controls (HC) and Crohn’s disease (CD) patients]. Solid horizontal lines indicate the group mean, and vertical lines represent ± 2 standard deviations (SD) used as the threshold for identifying low-quality spectra. [file Image_2.png]
